# Admission glucose, HbA1c levels and inflammatory cytokines in patients with acute ST-elevation myocardial infarction

**DOI:** 10.1186/s12014-025-09530-y

**Published:** 2025-02-17

**Authors:** Meisinger Christa, Freuer Dennis, Raake Philip, Linseisen Jakob, Schmitz Timo

**Affiliations:** 1 Epidemiology, Medical Faculty, University of Augsburg, University Hospital Augsburg, Stenglinstraße 2, 86156 Augsburg, Germany; 2 Department of Cardiology, Respiratory Medicine and Intensive Care, University Hospital Augsburg, Stenglinstraße 2, 86156 Augsburg, Germany

**Keywords:** Inflammatory plasma proteins, Cytokines, Glucose metabolism, Admission blood glucose, HbA1c, Myocardial infarction

## Abstract

**Background:**

To investigate the association between admission glucose and HbA1c values and inflammatory plasma proteins in hospitalized patients with acute ST-elevation myocardial infarction (STEMI).

**Methods:**

This analysis was based on 345 STEMI patients recorded by the population-based Myocardial Infarction Registry Augsburg between 2009 and 2013. Using the OLINK inflammatory panel, a total of 92 protein biomarkers were measured in arterial blood samples, which were obtained within the scope of cardiac catheterization immediately after admission. The associations between admission glucose and HbA1c levels and the 92 protein markers were investigated using multivariable linear regression models.

**Results:**

Admission glucose showed significantly positive associations with the inflammatory markers IL-10, IL-8, IL-6, FGF-21, IL-7, ST1A1, MCP-1, 4E-BP1, SIRT2, STAMBP and IL-18R1 after Bonferroni adjustment. HbA1c values were only significantly associated with IL-18R1. In stratified analyses, admission glucose was not significantly associated with any plasma protein in the diabetes subgroup, while there were several protein markers that showed significantly positive associations with admission glucose in STEMI patients without known diabetes, namely IL-10, CCL20, IL-8, MCP-1 and IL-6.

**Conclusions:**

Admission glucose in patients hospitalized due to an acute STEMI seems to be related to an inflammatory and immune-related response, expressed by an increase in inflammation-related plasma proteins in particular in non-diabetic patients with stress hyperglycemia. The present results may open new avenues for the development of biomarkers suitable as potential diagnostic or prognostic clinical markers.

**Supplementary Information:**

The online version contains supplementary material available at 10.1186/s12014-025-09530-y.

## Introduction

Stress hyperglycemia, a transient elevation of blood glucose, is common in critically ill patients, and the metabolic milieu in which stress induced hyperglycemia develops in the absence of pre-existing diabetes mellitus is complex [1]. A combination of hormonal and metabolic changes due to acute stress, including the presence of excessive counter regulatory hormones, such as glucagon, epinephrine, cortisol, and growth hormone seems to play an important role [2]. Consequently, these changes affect carbohydrate metabolism and lead to increased gluconeogenesis associated with hepatic insulin resistance, which appears to be the main factor in hyperglycemia [3]. There is evidence that stress hyperglycemia can cause oxidative stress and the formation of pro-inflammatory cytokines [4, 5].

A high number of prior studies reported an association between acute hyperglycemia at admission and worse outcomes in patients with acute myocardial infarction (AMI) [6–11]. In particular in non-diabetic compared to diabetic AMI patients elevated admission glucose levels were related to short- and long-term mortality [7, 12], suggesting that acutely elevated glucose levels, rather than chronically elevated glucose levels, may be the cause of a poorer outcome. To date, no clear explanation exists, why stress hyperglycemia was mostly associated with a higher risk of mortality in AMI patients without than with diabetes.

There are prior studies on this issue investigating the association between stress hyperglycemia and selected inflammatory markers in AMI patients [13, 14]. For example, it was shown recently that MCP-1 may play an important role regarding the inflammatory response due to stress hyperglycemia in STEMI patients [14]. The aim of the present study was therefore to comprehensively identify inflammatory and immune-related biomarkers associated with admission glucose and HbA1c in diabetic and non-diabetic patients hospitalized with STEMI.

## Methods

### Study population

The present analysis was based on data from the population-based Augsburg Myocardial Infarction Registry, Germany. It was established in 1984 as a part of the MONICA-project (Monitoring Trends and Determinants in Cardiovascular disease) and has been operated as KORA (Cooperative Health Research in the Augsburg Region) Myocardial Infarction Registry since 1996. The study area comprises approximately 680,000 inhabitants (city of Augsburg, and the two adjacent counties). All acute myocardial infarction (AMI) patients admitted to one out of eight hospitals in the study area were consecutively registered on condition the patient was between 25 and 84 years of age and had his primary residence in the study region at the time of the infarction. Detailed information on case identification, diagnostic classification of events and quality control of the data can be found in previous publications [15, 16].

The specific blood samples used for the present study derived from patients with STEMI admitted to the University hospital of Augsburg between May, 2009, and July, 2013. For 398 consecutive patients, a panel of 92 inflammatory plasma proteins was measured (mean age: 63.5 years (SD: 11.9), male: 73.1%). There was missing information on plasma proteins for 3 patients, for another 28 patients there was no information on either admission glucose or HbA1c levels and there were 22 patients with unknown BMI. Finally, a total of 345 AMI cases were considered for the present analysis.

All study participants gave written informed consent. Methods of data collection were approved by the ethics committee of the Bavarian Medical Association (Bayerische Landesärztekammer) and the study was performed in accordance with the Declaration of Helsinki.

### Data collection

All patients were interviewed during their hospital stay by trained study nurses using a standardized questionnaire. In addition to this, the patients’ medical chart was reviewed. In this way, a variety of important data (including demographic data, data on cardiovascular risk factors, medical history, comorbidities [including diabetes], medication, laboratory parameters, and electrocardiography) was collected from each patient. A patient was categorized as suffering from diabetes if there was either a former physician diagnosis of diabetes mellitus or the patients HbA1c was above 6.5% (48 mmol/mol).

The blood samples for the 398 patients were obtained within the scope of cardiac catheterization, which was mostly performed immediately after hospital admission. Therefore, EDTA blood samples (arterial blood) were taken right at the beginning of the catheterization. The samples were then immediately processed in the catheter laboratory (centrifugation, aliquoting, and freezing at -80 °C).

### Clinical chemistry measurement

The Proseek^®^ Multiplex Inflammation panel (developed by Olink Proteomics, Uppsala, Sweden) was used for the measurement of the 92 plasma proteins. The measurements were based on the Proximity Extension Assay (PEA); detailed information on the process of measurement can be found directly at the website of Olink Proteomics [17] and in a previous publication [18]. A list of all measured plasma proteins including the short- and long-form names can be found in Table S1 of the supplementary material. All plasma proteins with 25% or more values below the limit of detection (LOD) were not considered for this analysis. For all other plasma proteins, we used the values provided by OLINK even if the value was below the LOD.

HbA1c levels were also determined in the blood samples derived from the catheterization using a reverse-phase cation-exchange high-pressure liquid chromatography (HPLC) method (Analyzer HA 8160; Menarini, Florence, Italy). All other blood parameters including admission glucose were measured in venous blood samples taken at hospital admission or during hospital stay as part of the regular diagnosis and routine treatment.

### Statistical analysis

Categorical variables were compared using Chi-square tests and the results were presented as absolute frequencies with percentages. For the comparison of continuous variables Student’s t tests (in case of normal distribution) and Mann–Whitney U tests (for non-normally distributed variables) were used. The corresponding results were presented as mean and SD (standard deviation) or median and inter-quartile range (IQR).

### Linear regression models

First, the obtained values for each plasma protein were standardized (the variable was centered and normalized so that the transformed variable had an expectancy value of 0 and a statistical variance of 1), which provides comparability between the 92 plasma proteins. Linear regression models were calculated to examine the associations between the inflammatory plasma proteins (outcome) and parameters of glucose metabolism (admission glucose and HbA1c levels; exposures). According to literature research, the models were adjusted for sex (male/female), age (in years), renal function according to estimated GFR (3 groups: eGFR ≥ 60 ml/min/1,73m^2^, eGFR 30–59 ml/min/1,73m^2^, eGFR < 30 ml/min/1,73m^2^), diabetes mellitus (yes/no) and BMI (continuous). Bonferroni adjustment of the obtained *p*-values was conducted to control for multiple testing. Observations with Cook’s distance values > 0.5 were eliminated from the regression models as this indicates excessively influential data points. The effect estimates (β-coefficient and 95% CI) of the linear models must be interpreted as the expected change in standardized outcome associated with a one unit increase in the exposure variable (one mg/dl for admission glucose and 1% for HbA1c).

In a subsequent analysis, we calculated linear regression models separately for patients with and without diabetes to analyze the association between the inflammatory plasma proteins and admission glucose and HbA1c levels in each of the two subgroups. These models were adjusted for sex, age, renal function and BMI.

## Results

Table 1 displays the baseline characteristics for the total sample and stratified for diabetes. STEMI patients with known diabetes were older, had more frequently hypertension, more often low left ventricular EF values, higher admission glucose, HbA1c, and peak CRP values.

**Table 1:** Baseline characteristics for the total sample and by history of diabetes (mean, SD; median IQR; *n* (%))

	Total sample (*n* = 345)	Patients with a diagnosis of diabetes (*n* = 106)	Patients with no diagnosis of diabetes (*n* = 239)	*p*-value	N*
Age (mean, SD)	63.2 (11.9)	65.3 (11.5)	62.2 (12.0)	0.022	345
Male	251 (72.8)	81 (76.4)	170 (71.1)	0.375	345
**Comorbidities**					
Hypertension	260 (75.4)	93 (87.7)	167 (69.9)	0.001	345
Hyperlipidemia	196 (56.8)	69 (65.1)	127 (53.1)	0.051	345
Smoking status				0.236	334
Current smoker	138 (41.3)	36 (34.6)	102 (44.3)		
Ex-smoker	95 (28.4)	32 (30.8)	63 (27.4)		
Never smoker	101 (30.2)	36 (34.6)	65 (28.3)		
**Clinical characteristics**					
Prehospital time in minutes (median, IQR)	120.0 (71.0–271.0)	125.5 (60.0–410.8)	119.0 (80.0–255.0)	0.77	325
Left ventricular EF				0.025	332
> 50%	161 (48.5)	45 (42.9)	116 (51.1)		
41–50%	78 (23.5)	28 (26.7)	50 (22.0)		
31–40%	75 (22.6)	21 (20.0)	54 (23.8)		
≤ 30%	18 (5.4)	11 (10.5)	7 (3.1)		
Kidney function				0.727	345
eGFR ≥ 60 ml/min/1.73 m^2^	245 (71.0)	75 (70.8)	170 (71.1)		
eGFR ≥ 60 ml/min/1.73 m^2^	90 (26.1)	29 (27.4)	61 (25.5)		
eGFR ≥ 60 ml/min/1.73 m^2^	10 (2.9)	2 (1.9)	8 (3.3)		
**Treatment**					
PCI	319 (92.5)	95 (89.6)	224 (93.7)	0.267	345
Bypass therapy	34 (9.9)	14 (13.2)	20 (8.4)	0.232	345
Lysis therapy	3 (0.9)	--	3 (1.3)		345
**Laboratory values** (median, IQR)					
Admission glucose in mg/dl	137.0 (116.0–167.0)	177.0 (149.2–221.8)	125.0 (112.0–149.0)	< 0.001	345
HbA1c in%	5.8 (5.5–6.3)	6.7 (6.1–7.3)	5.6 (5.4–5.9)	< 0.001	345
HbA1c in mmol/mol	39.9 (36.6–45.4)	49.7 (43.1–56.3)	37.7 (35.5–41.0)	< 0.001	345
Troponin I at admission in µg/ml	0.5 (0.1–5.8)	0.6 (0.1–8.1)	0.5 (0.1–3.8)	0.162	339
peak CRP in mg/dl	0.4 (0.3–1.0)	0.6 (0.3–1.3)	0.3 (0.3–0.8)	0.001	343

* number of cases with valid information

**Fig. 1:**
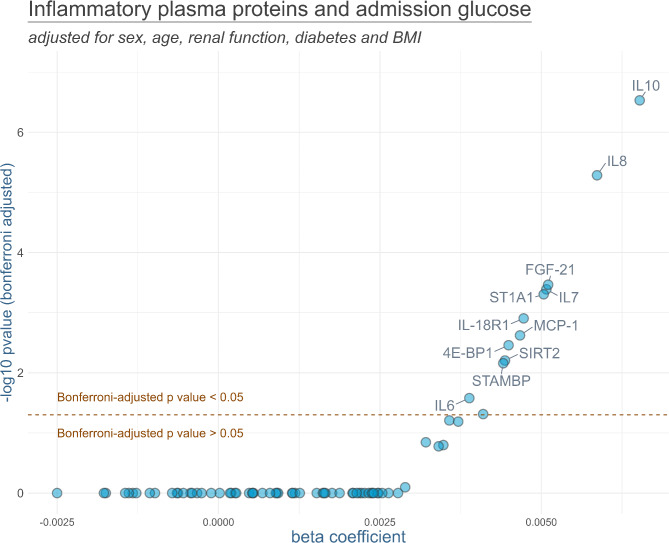
Results of the linear regression models analyzing the association between admission glucose and inflammatory plasma proteins. The models were adjusted for sex, age, renal function, diabetes and BMI. *P*-values were Bonferroni-adjusted. Names of the markers are presented for all markers with Bonferroni-adjusted *p*-values below 0.05

**Fig. 2:**
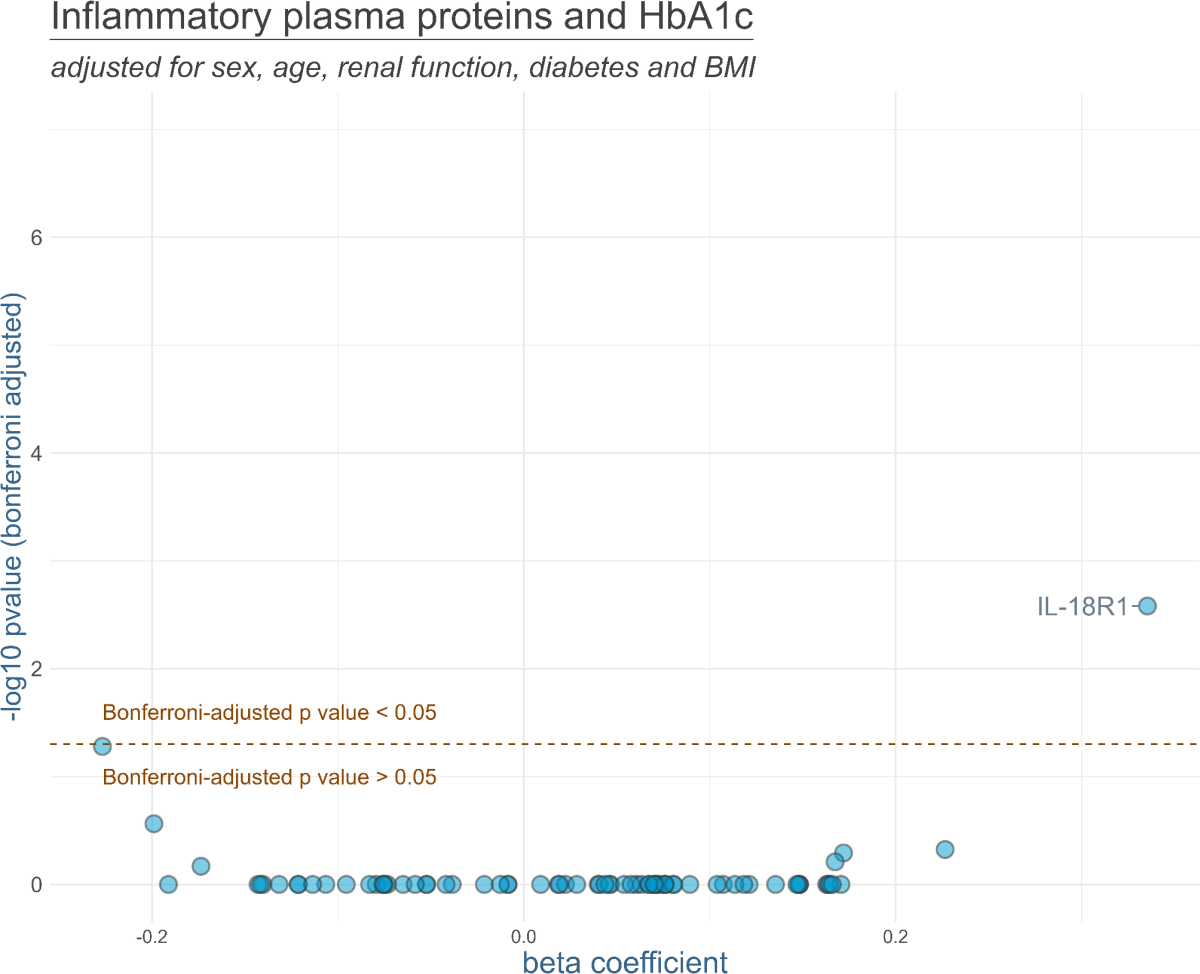
Results of the linear regression models analyzing the association between HbA1c and inflammatory plasma proteins. The models were adjusted for sex, age, renal function, diabetes and BMI. *P*-values were Bonferroni-adjusted. Names of the markers are presented for all markers with Bonferroni-adjusted *p*-values below 0.05

Results of the linear regression models are displayed in Fig. 1 (admission glucose) and Fig. 2 (HbA1c). For admission glucose, the following plasma proteins showed a positively significant association after Bonferroni adjustment of *p*-values: interleukin-10 (IL-10), interleukin-8 (IL-8), fibroblast growth factor 21 (FGF-21), interleukin-7 (IL-7), Sulfotransferase 1A1 (ST1A1), interleukin-18 receptor 1 (IL-18R1), monocyte chemoattractant protein-1 (MCP-1), Eukaryotic translation initiation factor 4E-binding protein 1 (4E-BP1), sirtuin-2 (SIRT2), STAM-binding protein (STAMBP), and interleukin-6 (IL-6). HbA1c values were only significantly associated with IL-18R1.

In Table S1 of the supplementary material, the total results (beta coefficient, 95% CI, Bonferroni-adjusted *p* value) are displayed in table form. This table also provides the full names of the measured plasma proteins.

**Fig. 3:**
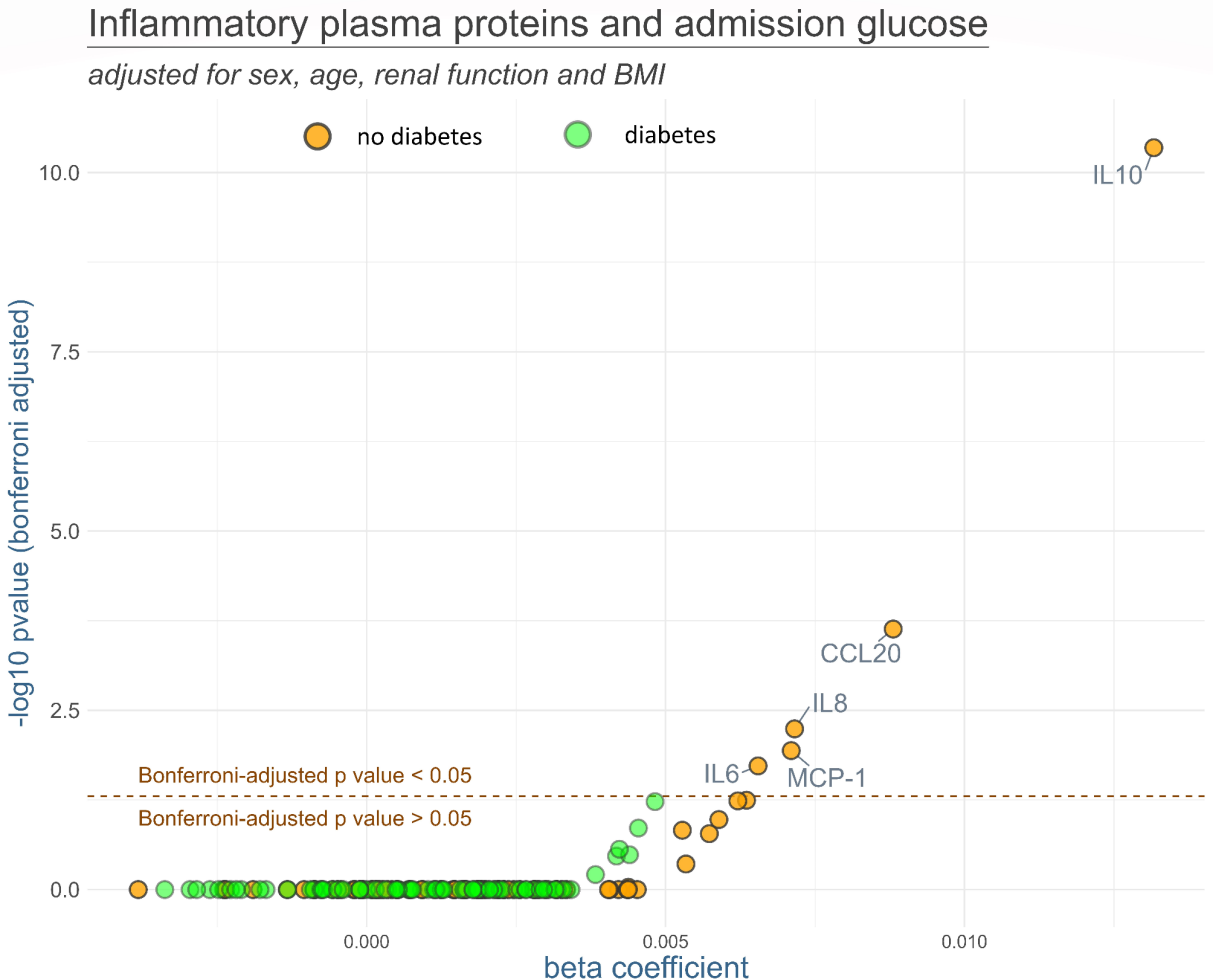
Results of the linear regression models analyzing the association between inflammatory plasma proteins and admission glucose separately for patients with diabetes (green) and non-diabetes patients (orange). The models were adjusted for sex, age, renal function and BMI. *P*-values were Bonferroni-adjusted. Names of the markers are presented for all markers with Bonferroni-adjusted *p*-values below 0.05

In stratified analyses, admission glucose was not significantly associated with any plasma protein in the diabetes subgroup, while there were several protein markers that showed significantly positive associations with admission glucose in STEMI patients without known diabetes, namely IL-10, CCL20, IL-8, MCP-1 and IL-6. The results of the stratified (diabetes and no diabetes) linear regression models for admission glucose are presented in Fig. 3.

## Discussion

The present study investigated the associations between admission glucose and HbA1c levels and inflammatory proteins in patients admitted to hospital due to an acute STEMI. Admission glucose was associated with a number of inflammatory markers in the total sample. In stratified analyses, in non-diabetic AMI patients stress hyperglycemia at admission was independently associated with the cytokines IL-6, IL-8, IL-10, CCL20, and MCP-1. Contrary, admission glucose in patients with known diabetes or long-term glucose control as assessed by HbA1c levels were not independently associated with inflammatory markers.

## Inflammatory responses to admission glucose in the total sample

With increasing admission glucose, a number of inflammatory biomarkers were elevated in STEMI patients, including common markers such as IL-6, IL-8, IL-10, interleukin-18 receptor 1 (IL-18R1), and Fibroblast growth factor 21 (FGF-21). FGF21 is a metabolic regulator that, among other effects, increases insulin sensitivity and thus lowers blood sugar levels [19]; it has been found to play an important role in the prognosis after STEMI [20]. In the present study, higher admission glucose went along with an increase in interleukins, such as IL-6, which are known to be elevated in AMI patients independent from glucose levels [21]. However, to date there has been a lack of studies in this patient group on the extent to which admission glucose plays a role in this context. The present results confirmed the results from a recent study on STEMI patients undergoing primary percutaneous coronary intervention, which showed that stress hyperglycemia and diabetes may contribute to high levels of the chemokine MCP-1 [14]. Other studies reported, that serum MCP-1 was significantly elevated in patients with type 2 diabetes and that oxidative stress increases locally produced MCP-1, which triggers macrophage-induced inflammation [22, 23].

In the present investigation, increased levels of further inflammatory markers were identified, that so far were not reported by other studies on this issue, such as ST1A1, SIRT2, and STAMBP.

STAMBP is a deubiquitinating protein which has been identified as a potential diagnostic biomarker for early Alzheimer’s disease [24], fibromyalgia [25] and squamous cell carcinoma of the esophagus [26]. The sulfotransferase ST1A1 catalyzes the sulfation of neurotransmitters, catecholamines, estrogens, and phenolics [27]. Such as STAMBP, ST1A1 was also associated with an increased risk of esophageal squamous cell carcinoma in prior investigations [26]. Furthermore, a recent study from our group found that changes in ST1A1 expression predicted short-term mortality in STEMI patients [20]. SIRT2, a deacetylase that is mainly localized in the cytoplasm, seems to play an important role in biological processes, for example neuronal differentiation, mitotic regulation, cell homeostasis, ageing, and oxidative stress [28].

In addition, we found a strong association between admission glucose and the eukaryotic translation initiation factor 4E (eIF4E)-binding protein 1 (4E-BP1). The translation repressor 4E-BP1 is a known substrate of the mTOR (Mechanistic Target of Rapamycin) signaling pathway, which regulates important cellular processes and is involved in several pathological conditions, including type 2 diabetes, obesity and cancer [29].

### Association between HbA1c levels and inflammatory markers in STEMI patients

HbA1c at hospital admission due to STEMI was only independently associated with increased levels of the interleukin-18 receptor 1 (IL-18R1), which is the receptor for IL-18 and essential for IL-18 mediated signal transduction. So far, the role of IL-18 in glucose metabolism is inconsistent. Although IL-18 has been ascribed a positive role in glucose homeostasis [30, 31], a number of clinical studies have observed an upregulation of circulating IL-18 in patients with type 2 diabetes [32, 33], independent of risk factors such as BMI and the presence of a generalized pro-inflammatory state [34]. Other investigations reported a correlation between circulating IL-18 levels and the Homeostasis Model Assessment of Insulin Resistance (HOMA-IR) index [35] or glucose intolerance independent of BMI or age [36, 37]. Conversely, a decrease in IL-18 was associated with an improvement in β-cell function in individuals suffering from type 2 diabetes [38].

### Inflammatory responses to admission glucose in non-diabetic and diabetic STEMI patients

In the non-diabetic group, admission blood glucose levels were related to chemokines such as IL-8, IL-6, IL-10 and MCP-1 [39, 40]. IL-10 is a Th2-type cytokine that is produced by a variety of immunological cell types and has the ability to inhibit the production and activity of cytokines, such as IL-6, thereby exerting an anti-inflammatory effect [41]. However, there is also evidence that excess IL-10 may be associated with poorer clinical outcomes [42]. Prior studies have shown that IL-10 can increase insulin sensitivity and inhibit the negative effects of inflammatory cytokines on insulin signaling and glucose homeostasis [43]. Possibly, the found association between admission glucose and IL-10 could be explained as a counter-regulatory response to insulin resistance and stress hyperglycemia due to acute illness [44].

Furthermore, this study showed higher levels of CCL20 with increasing stress hyperglycemia [45, 46]. CCL20 is a chemokine that plays a key role in the regulation of dendritic cell trafficking and the recruitment and activation of T cells [47]. It is produced by activated cells such as monocytes, T cells, endothelial cells and fibroblasts. These results are in accordance with previous findings demonstrating that CCL20 is upregulated in response to high glucose [46].

Interestingly, in diabetic STEMI patients no inflammatory markers remained significantly associated with admission glucose after Bonferroni adjustment.

The current results indicated that especially in non-diabetic STEMI patients, the presence of stress hyperglycemia is associated with inflammatory markers. This may partly explain why non-diabetic AMI patients with elevated admission glucose have a worse prognosis than diabetic AMI patients [48].

### Strengths and limitation

The current study has several strengths. First, the analyses were based on data from the population-based Augsburg Myocardial Infarction Registry with consecutive recruitment, which minimizes the effect of selection bias. The highly standardized collection of the arterial blood samples was done immediately before the PCI procedure and the samples were processed and stored immediately afterwards. Finally, a large amount of additional information on the patients was available, which could be considered as confounders in the linear regression models.

The present analysis has also some limitations. No studies were available to validate our finding for the identified protein markers associated with admission glucose or HbA1c. Furthermore, due to the cross-sectional design of this study no conclusions about causality could be drawn (including the possibility of reverse causality). Also, residual or unmeasured confounding cannot be excluded. Finally, this study included only STEMI patients aged between 25 and 84 years, thus the results may not be generalized to all ethnicities as well as to Non-ST-elevation infarctions.

## Conclusions

In summary, a number of inflammatory markers were associated with admission glucose in STEMI patients. The found associations between admission glucose and inflammatory proteins, which belong to inflammatory and immune-related signaling pathways, point towards a stress hyperglycemia-induced inflammation in non-diabetic patients. Further studies are needed to clarify the pathophysiological mechanisms involved. The present results may open new avenues for the development of biomarkers suitable as potential diagnostic or prognostic clinical markers.

## Electronic supplementary material

Below is the link to the electronic supplementary material.

Supplementary Material 1

## Data Availability

The datasets generated during and/or analyzed in the current study are not publicly available due to data protection aspects but are available in an anonymized form from the corresponding author on reasonable request.

## Abbreviations

AMI: Acute myocardial infarction
BMI: Body mass index
CI: Confidence interval
CRP: C-reactive protein
EF: Ejection fraction
GFR: Glomerular filtration rate
HPLC: High-pressure liquid chromatography
HOMA-IR: Homeostasis model assessment of insulin resistance
IQR: Interquartile range
KORA: Cooperative health research in the augsburg region
LOD: Limit of detection
MONICA: Monitoring trends and determinants in cardiovascular disease
PEA: Proximity extension assay
mTOR: Mechanistic target of rapamycin
SD: Standard deviation
STEMI: ST-elevation infarctions
PCI: Percutaneous coronary intervention
