# Supplementary material for: Admission glucose, HbA1c levels and inflammatory cytokines in patients with acute ST-elevation myocardial infarction
Source: Clin Proteomics. 2025 Feb 17;22:8. doi: 10.1186/s12014-025-09530-y (PMC11834255; doi:10.1186/s12014-025-09530-y)
Supplement: Supplementary file 1 — Supplementary Material 1 [file 12014_2025_9530_MOESM1_ESM.docx]

**Admission glucose, HbA1c levels and inflammatory cytokines in patients with acute ST-elevation myocardial infarction**

Meisinger C^1^, Freuer D^1^, Raake P^2^, Linseisen J^1^, Schmitz T^1^

***Table S1:*** Results of the linear regression models

|  |  |  | **Admission glucose - total sample** | | **HbA1c - total sample** | | **Admission glucose - diabetes patients** | | **Admission glucose - no diabetes patients** | |
| --- | --- | --- | --- | --- | --- | --- | --- | --- | --- | --- |
| **paramter (short name)** | **paramter (long name)** | **Number of values below LOD** | **beta (95%CI)** | **P value** | **beta (95%CI)** | **P value** | **beta (95%CI)** | **P value** | **beta (95%CI)** | **P value** |
| 4E-BP1 | Eukaryotic translation initiation factor 4E-binding protein 1 (4E-BP1) | 0.73% | 0.0045 [0.0024,0.0066] | 0.004 | 0.0673 [-0.0644,0.1989] | 1.000 | 0.0034 [0.0007,0.0062] | 1.000 | 0.0042 [0.0004,0.008] | 1.000 |
| ADA | Adenosine Deaminase (ADA) | 0.73% | 0.0024 [0.0002,0.0046] | 1.000 | 0.0577 [-0.077,0.1924] | 1.000 | -0.0007 [-0.0035,0.0021] | 1.000 | 0.0032 [-0.0003,0.0067] | 1.000 |
| ARTN | Artemin (ARTN) | 18.78% | -0.0007 [-0.003,0.0015] | 1.000 | -0.1136 [-0.2491,0.022] | 1.000 | -0.0009 [-0.004,0.0021] | 1.000 | 0.0004 [-0.0034,0.0042] | 1.000 |
| AXIN1 | Axin-1 (AXIN1) | 2.68% | 0.0022 [0,0.0045] | 1.000 | 0.1706 [0.0054,0.3358] | 1.000 | 0.0022 [-0.0006,0.0051] | 1.000 | 0.0026 [-0.0013,0.0064] | 1.000 |
| Beta-NGF | Beta-nerve growth factor (Beta-NGF) | 98.78% |  |  |  |  |  |  |  |  |
| CASP-8 | Caspase-8 (CASP-8 ) | 1.22% | 0.0037 [0.0015,0.0059] | 0.079 | 0.0191 [-0.1136,0.1518] | 1.000 | 0.0018 [-0.001,0.0045] | 1.000 | 0.0045 [0.0007,0.0083] | 1.000 |
| CCL11 | Eotaxin (CCL11) | 0.73% | 0.0025 [0.0003,0.0047] | 1.000 | 0.1135 [-0.0197,0.2467] | 1.000 | 0.0032 [0.0003,0.0061] | 1.000 | 0.0019 [-0.0018,0.0055] | 1.000 |
| CCL19 | C-C motif chemokine 19 (CCL19) | 0.73% | 0.0005 [-0.0017,0.0026] | 1.000 | 0.0605 [-0.0691,0.1902] | 1.000 | -0.0006 [-0.0037,0.0025] | 1.000 | 0.0028 [-0.0007,0.0063] | 1.000 |
| CCL20 | C-C motif chemokine 20 (CCL20) | 0.73% | 0.0034 [0.0012,0.0056] | 0.205 | -0.0126 [-0.1452,0.12] | 1.000 | 0.0005 [-0.0023,0.0032] | 1.000 | 0.0088 [0.0052,0.0124] | <0.001 |
| CCL23 | C-C motif chemokine 23 (CCL23) | 0.73% | 0.0008 [-0.0013,0.0029] | 1.000 | 0.0632 [-0.064,0.1904] | 1.000 | 0.0003 [-0.0025,0.0031] | 1.000 | 0.0016 [-0.0019,0.0052] | 1.000 |
| CCL25 | C-C motif chemokine 25 (CCL25) | 0.73% | 0.0021 [0,0.0042] | 1.000 | 0.0436 [-0.0829,0.1702] | 1.000 | 0.0012 [-0.0012,0.0035] | 1.000 | 0.0044 [0.0007,0.008] | 1.000 |
| CCL28 | C-C motif chemokine 28 (CCL28) | 2.44% | -0.0018 [-0.0039,0.0004] | 1.000 | -0.1429 [-0.2716,-0.0143] | 1.000 | -0.0028 [-0.0057,0.0001] | 1.000 | 0.0009 [-0.0027,0.0045] | 1.000 |
| CCL3 | C-C motif chemokine 3 (CCL3) | 0.73% | 0.0026 [0.0005,0.0047] | 1.000 | 0.0285 [-0.0996,0.1566] | 1.000 | 0.0007 [-0.0019,0.0034] | 1.000 | 0.0059 [0.0023,0.0095] | 0.129 |
| CCL4 | C-C motif chemokine 4 (CCL4 ) | 0.73% | 0.0035 [0.0013,0.0057] | 0.195 | 0.0729 [-0.0616,0.2075] | 1.000 | 0.0025 [-0.0003,0.0053] | 1.000 | 0.0053 [0.0016,0.0091] | 0.539 |
| CD244 | Natural killer cell receptor 2B4 (CD244) | 0.73% | 0.0012 [-0.001,0.0034] | 1.000 | -0.0212 [-0.1523,0.1098] | 1.000 | 0.0011 [-0.0019,0.0042] | 1.000 | 0.0009 [-0.0026,0.0045] | 1.000 |
| CD40 | CD40L receptor (CD40) | 0.73% | 0.0016 [-0.0002,0.0035] | 1.000 | -0.0087 [-0.1214,0.1041] | 1.000 | 0.0012 [-0.0013,0.0038] | 1.000 | 0.0019 [-0.0011,0.0049] | 1.000 |
| CD5 | T-cell surface glycoprotein CD5 (CD5) | 0.73% | -0.0005 [-0.0027,0.0016] | 1.000 | -0.0955 [-0.2219,0.031] | 1.000 | 0.0007 [-0.002,0.0034] | 1.000 | -0.0019 [-0.0055,0.0017] | 1.000 |
| CD6 | T cell surface glycoprotein CD6 isoform (CD6) | 0.73% | -0.001 [-0.0032,0.0012] | 1.000 | -0.0757 [-0.2081,0.0567] | 1.000 | -0.0009 [-0.004,0.0022] | 1.000 | -0.0009 [-0.0044,0.0027] | 1.000 |
| CD8A | T-cell surface glycoprotein CD8 alpha chain (CD8A) | 0.73% | -0.0006 [-0.0028,0.0016] | 1.000 | -0.0522 [-0.1856,0.0813] | 1.000 | 0.0005 [-0.0023,0.0033] | 1.000 | 0.0002 [-0.0038,0.0041] | 1.000 |
| CDCP1 | CUB domain-containing protein 1 (CDCP1) | 0.73% | 0.0022 [0.0001,0.0043] | 1.000 | 0.1675 [0.0436,0.2914] | 0.757 | 0.0018 [-0.0007,0.0044] | 1.000 | 0.0016 [-0.002,0.0052] | 1.000 |
| CSF-1 | Macrophage colony-stimulating factor 1 (CSF-1) | 0.73% | -0.0011 [-0.0031,0.001] | 1.000 | 0.0702 [-0.0816,0.2219] | 1.000 | -0.0004 [-0.0038,0.0031] | 1.000 | -0.0024 [-0.0052,0.0005] | 1.000 |
| CST5 | Cystatin D (CST5) | 0.73% | 0.0019 [0,0.0038] | 1.000 | -0.0385 [-0.1804,0.1034] | 1.000 | 0.0001 [-0.0022,0.0025] | 1.000 | -0.0001 [-0.0034,0.0033] | 1.000 |
| CX3CL1 | Fractalkine (CX3CL1 ) | 0.73% | 0.0025 [0.0005,0.0045] | 1.000 | 0.009 [-0.1128,0.1309] | 1.000 | 0.0032 [0.0002,0.0061] | 1.000 | 0.0019 [-0.0012,0.0049] | 1.000 |
| CXCL1 | C-X-C motif chemokine 1 (CXCL1) | 0.73% | 0.0021 [-0.0001,0.0042] | 1.000 | 0.1073 [-0.0233,0.238] | 1.000 | 0.0023 [-0.0007,0.0052] | 1.000 | 0.002 [-0.0016,0.0056] | 1.000 |
| CXCL10 | C-X-C motif chemokine 10 (CXCL10 ) | 0.98% | 0.0002 [-0.0019,0.0022] | 1.000 | -0.0749 [-0.1989,0.0491] | 1.000 | -0.0018 [-0.0044,0.0009] | 1.000 | 0.0033 [-0.0002,0.0067] | 1.000 |
| CXCL11 | C-X-C motif chemokine 11 (CXCL11) | 0.73% | 0.0018 [-0.0003,0.0039] | 1.000 | 0.0893 [-0.037,0.2156] | 1.000 | 0.0013 [-0.0016,0.0041] | 1.000 | 0.0023 [-0.0012,0.0058] | 1.000 |
| CXCL5 | C-X-C motif chemokine 5 (CXCL5 ) | 0.73% | 0.0015 [-0.0007,0.0037] | 1.000 | 0.1469 [0.0163,0.2776] | 1.000 | 0.0019 [-0.0013,0.0051] | 1.000 | 0.0002 [-0.0033,0.0037] | 1.000 |
| CXCL6 | C-X-C motif chemokine 6 (CXCL6) | 0.73% | 0.0017 [-0.0005,0.0038] | 1.000 | 0.0405 [-0.0893,0.1704] | 1.000 | 0.001 [-0.002,0.0041] | 1.000 | 0.003 [-0.0005,0.0065] | 1.000 |
| CXCL9 | C-X-C motif chemokine 9 (CXCL9 ) | 0.73% | -0.0003 [-0.0023,0.0018] | 1.000 | -0.1403 [-0.2627,-0.0178] | 1.000 | -0.0023 [-0.0047,0.0002] | 1.000 | 0.0033 [-0.0002,0.0069] | 1.000 |
| DNER | Delta and Notch-like epidermal growth factor-related receptor (DNER) | 0.73% | -0.0014 [-0.0035,0.0008] | 1.000 | 0.0807 [-0.0771,0.2384] | 1.000 | -0.0008 [-0.004,0.0025] | 1.000 | -0.0038 [-0.0071,-0.0006] | 1.000 |
| EN-RAGE | Protein S100-A12 (EN-RAGE ) | 0.73% | 0.0029 [0.0007,0.0051] | 0.981 | 0.1184 [-0.0154,0.2522] | 1.000 | 0.0021 [-0.0008,0.005] | 1.000 | 0.0024 [-0.0015,0.0063] | 1.000 |
| FGF-19 | Fibroblast growth factor 19 (FGF-19) | 0.73% | 0 [-0.0022,0.0022] | 1.000 | -0.1911 [-0.3514,-0.0308] | 1.000 | 0.0008 [-0.0019,0.0035] | 1.000 | 0.0002 [-0.0035,0.004] | 1.000 |
| FGF-21 | Fibroblast growth factor 21 (FGF-21) | 0.73% | 0.0051 [0.003,0.0073] | <0.001 | 0.2266 [0.0644,0.3888] | 0.582 | 0.0038 [0.001,0.0066] | 0.758 | 0.0064 [0.0027,0.01] | 0.070 |
| FGF-23 | Fibroblast growth factor 23 (FGF-23) | 62.68% |  |  |  |  |  |  |  |  |
| FGF-5 | Fibroblast growth factor 5 (FGF-5) | 5.12% | 0.0003 [-0.002,0.0025] | 1.000 | -0.0524 [-0.1883,0.0834] | 1.000 | -0.0017 [-0.0047,0.0014] | 1.000 | 0.0032 [-0.0006,0.0069] | 1.000 |
| Flt3L | Fms-related tyrosine kinase 3 ligand (Flt3L) | 0.73% | 0.0005 [-0.0018,0.0029] | 1.000 | -0.1316 [-0.2658,0.0026] | 1.000 | -0.0001 [-0.0038,0.0036] | 1.000 | 0.0017 [-0.0018,0.0051] | 1.000 |
| GDNF | Glial cell line-derived neurotrophic factor (GDNF) | 12.44% | -0.0004 [-0.0026,0.0018] | 1.000 | -0.0794 [-0.212,0.0533] | 1.000 | -0.0026 [-0.0059,0.0006] | 1.000 | 0.0001 [-0.0035,0.0036] | 1.000 |
| HGF | Hepatocyte growth factor (HGF) | 0.73% | -0.0013 [-0.0035,0.001] | 1.000 | -0.1989 [-0.3326,-0.0653] | 0.336 | -0.0022 [-0.0054,0.001] | 1.000 | 0.0003 [-0.0034,0.0039] | 1.000 |
| IFN-gamma | Interferon gamma (IFN-gamma) | 1.22% | -0.0014 [-0.0036,0.0007] | 1.000 | -0.0584 [-0.1899,0.073] | 1.000 | -0.0013 [-0.0045,0.0019] | 1.000 | 0.0012 [-0.0024,0.0049] | 1.000 |
| IL-1 alpha | Interleukin-1 alpha (IL-1 alpha) | 94.63% |  |  |  |  |  |  |  |  |
| IL10 | Interleukin-10 (IL10) | 0.73% | 0.0065 [0.0044,0.0086] | <0.001 | -0.0734 [-0.2074,0.0607] | 1.000 | 0.0029 [0.0001,0.0057] | 1.000 | 0.0132 [0.0098,0.0166] | <0.001 |
| IL-10RA | Interleukin-10 receptor subunit alpha (IL-10RA) | 37.56% |  |  |  |  |  |  |  |  |
| IL-10RB | Interleukin-10 receptor subunit beta (IL-10RB) | 0.73% | 0.0002 [-0.0018,0.0022] | 1.000 | 0.0699 [-0.077,0.2167] | 1.000 | 0.0005 [-0.0022,0.0032] | 1.000 | -0.0002 [-0.0035,0.0031] | 1.000 |
| IL-12B | Interleukin-12 subunit beta (IL-12B) | 0.73% | -0.0006 [-0.0027,0.0014] | 1.000 | -0.1213 [-0.2722,0.0296] | 1.000 | 0.0007 [-0.0024,0.0039] | 1.000 | -0.0001 [-0.0035,0.0033] | 1.000 |
| IL13 | Interleukin-13 (IL-13) | 91.71% |  |  |  |  |  |  |  |  |
| IL-15RA | Interleukin-15 receptor subunit alpha (IL-15RA) | 0.98% | 0.0005 [-0.0014,0.0025] | 1.000 | -0.0418 [-0.1598,0.0762] | 1.000 | 0.0016 [-0.0016,0.0047] | 1.000 | -0.001 [-0.004,0.0019] | 1.000 |
| IL-17A | Interleukin-17A (IL-17A) | 23.90% | 0.0009 [-0.0013,0.0032] | 1.000 | -0.0084 [-0.143,0.1262] | 1.000 | 0 [-0.0024,0.0024] | 1.000 | 0.0005 [-0.0034,0.0045] | 1.000 |
| IL-17C | Interleukin-17C (IL-17C) | 0.73% | -0.0004 [-0.0025,0.0017] | 1.000 | -0.0649 [-0.1918,0.0619] | 1.000 | -0.0025 [-0.005,0.0001] | 1.000 | 0.0019 [-0.0016,0.0054] | 1.000 |
| IL18 | Interleukin-18 (IL-18) | 0.73% | 0.0011 [-0.0011,0.0033] | 1.000 | 0.0185 [-0.1138,0.1509] | 1.000 | -0.0001 [-0.003,0.0028] | 1.000 | 0.0022 [-0.0015,0.0059] | 1.000 |
| IL-18R1 | Interleukin-18 receptor 1 (IL-18R1) | 0.73% | 0.0047 [0.0026,0.0069] | 0.002 | 0.3354 [0.1782,0.4927] | 0.003 | 0.0044 [0.0014,0.0074] | 0.401 | 0.0044 [0.001,0.0078] | 1.000 |
| IL2 | Interleukin-2 (IL-2) | 98.54% |  |  |  |  |  |  |  |  |
| IL-20 | Interleukin-20 (IL-20) | 92.93% |  |  |  |  |  |  |  |  |
| IL-20RA | Interleukin-20 receptor subunit alpha (IL-20RA) | 61.46% |  |  |  |  |  |  |  |  |
| IL-22 RA1 | Interleukin-22 receptor subunit alpha-1 (IL-22 RA1) | 93.17% |  |  |  |  |  |  |  |  |
| IL-24 | Interleukin-24 (IL-24) | 75.85% |  |  |  |  |  |  |  |  |
| IL-2RB | Interleukin-2 receptor subunit beta (IL-2RB) | 91.95% |  |  |  |  |  |  |  |  |
| IL33 | Interleukin-33 (IL-33) | 96.83% |  |  |  |  |  |  |  |  |
| IL4 | Interleukin-4 (IL-4) | 89.27% |  |  |  |  |  |  |  |  |
| IL5 | Interleukin-5 (IL5) | 82.44% |  |  |  |  |  |  |  |  |
| IL6 | Interleukin-6 (IL6) | 0.73% | 0.0039 [0.0018,0.006] | 0.032 | 0.0758 [-0.0535,0.2052] | 1.000 | 0.0022 [-0.0007,0.0051] | 1.000 | 0.0065 [0.0031,0.01] | 0.023 |
| IL7 | Interleukin-7 (IL-7) | 0.73% | 0.0051 [0.0029,0.0072] | <0.001 | 0.1637 [0.0309,0.2965] | 1.000 | 0.0045 [0.0017,0.0074] | 0.170 | 0.0057 [0.0021,0.0094] | 0.204 |
| IL8 | Interleukin-8 (IL-8) | 0.73% | 0.0059 [0.0038,0.008] | <0.001 | 0.1629 [0.0329,0.2929] | 1.000 | 0.0048 [0.0021,0.0076] | 0.073 | 0.0072 [0.0037,0.0107] | 0.007 |
| LAP TGF-beta-1 | Latency-associated peptide transforming growth factor beta-1 (LAP TGF-beta-1) | 0.73% | 0.0024 [0.0002,0.0046] | 1.000 | 0.1354 [0.0035,0.2673] | 1.000 | 0.0028 [-0.0001,0.0058] | 1.000 | 0.0015 [-0.0022,0.0051] | 1.000 |
| LIF | Leukemia inhibitory factor (LIF) | 76.59% |  |  |  |  |  |  |  |  |
| LIF-R | Leukemia inhibitory factor receptor (LIF-R) | 0.73% | 0.0036 [0.0015,0.0057] | 0.076 | 0.1479 [0.0215,0.2742] | 1.000 | 0.0042 [0.0013,0.007] | 0.418 | 0.0028 [-0.0006,0.0062] | 1.000 |
| MCP-1 | Monocyte chemotactic protein 1 (MCP-1) | 0.73% | 0.0047 [0.0025,0.0068] | 0.003 | 0.0467 [-0.0875,0.1809] | 1.000 | 0.003 [0.0003,0.0058] | 1.000 | 0.0071 [0.0035,0.0107] | 0.014 |
| MCP-2 | Monocyte chemotactic protein 2 (MCP-2) | 0.73% | 0.0041 [0.0018,0.0064] | 0.060 | 0.0459 [-0.0887,0.1805] | 1.000 | 0.0021 [-0.0013,0.0054] | 1.000 | 0.0062 [0.0026,0.0098] | 0.071 |
| MCP-3 | Monocyte chemotactic protein 3 (MCP-3) | 17.81% | 0.0025 [0.0003,0.0046] | 1.000 | 0.1482 [0.0199,0.2765] | 1.000 | 0.0021 [-0.0005,0.0048] | 1.000 | 0.0022 [-0.0014,0.0059] | 1.000 |
| MCP-4 | Monocyte chemotactic protein 4 (MCP-4) | 0.73% | 0.0021 [-0.0001,0.0043] | 1.000 | 0.104 [-0.0291,0.2371] | 1.000 | 0.0019 [-0.0008,0.0045] | 1.000 | 0.0015 [-0.0023,0.0053] | 1.000 |
| MMP-1 | Matrix metalloproteinase-1 (MMP-1) | 0.98% | 0.0028 [0.0006,0.005] | 1.000 | 0.0803 [-0.0542,0.2148] | 1.000 | 0.0027 [-0.0003,0.0056] | 1.000 | 0.0023 [-0.0015,0.006] | 1.000 |
| MMP-10 | Matrix metalloproteinase-10 (MMP-10) | 0.73% | 0.0005 [-0.0017,0.0027] | 1.000 | 0.04 [-0.0928,0.1727] | 1.000 | 0.0018 [-0.0008,0.0044] | 1.000 | -0.0013 [-0.0052,0.0026] | 1.000 |
| NRTN | Neurturin (NRTN) | 51.71% |  |  |  |  |  |  |  |  |
| NT-3 | Neurotrophin-3 (NT-3) | 18.54% | 0.0013 [-0.001,0.0035] | 1.000 | 0.0672 [-0.0669,0.2014] | 1.000 | 0.0013 [-0.0024,0.005] | 1.000 | -0.0009 [-0.0042,0.0024] | 1.000 |
| OPG | Osteoprotegerin (OPG) | 0.73% | 0.0032 [0.0012,0.0052] | 0.175 | 0.1212 [-0.0292,0.2716] | 1.000 | 0.002 [-0.0007,0.0048] | 1.000 | 0.0053 [0.002,0.0086] | 0.182 |
| OSM | Oncostatin-M (OSM) | 0.73% | 0.0023 [0,0.0045] | 1.000 | 0.1646 [0.0318,0.2974] | 1.000 | 0.0028 [0.0001,0.0055] | 1.000 | 0.0014 [-0.0024,0.0053] | 1.000 |
| PD-L1 | Programmed cell death 1 ligand 1 (PD-L1) | 0.73% | 0.0024 [0.0003,0.0044] | 1.000 | 0.0713 [-0.0523,0.195] | 1.000 | 0.0027 [-0.0003,0.0056] | 1.000 | 0.0016 [-0.0016,0.0049] | 1.000 |
| SCF | Stem cell factor (SCF) | 0.73% | -0.0013 [-0.0035,0.0008] | 1.000 | -0.0829 [-0.2395,0.0736] | 1.000 | -0.0021 [-0.0052,0.001] | 1.000 | 0.0007 [-0.0026,0.0041] | 1.000 |
| SIRT2 | SIR2-like protein 2 (SIRT2) | 12.20% | 0.0044 [0.0022,0.0066] | 0.008 | 0.0761 [-0.0583,0.2106] | 1.000 | 0.0033 [0.0005,0.0061] | 1.000 | 0.0041 [0.0002,0.008] | 1.000 |
| SLAMF1 | Signaling lymphocytic activation molecule (SLAMF1) | 1.22% | 0.0023 [0.0002,0.0045] | 1.000 | 0.071 [-0.0589,0.2008] | 1.000 | 0.0026 [-0.0002,0.0053] | 1.000 | 0.002 [-0.0017,0.0056] | 1.000 |
| ST1A1 | Sulfotransferase 1A1 (ST1A1) | 5.61% | 0.005 [0.0029,0.0072] | <0.001 | 0.054 [-0.0799,0.1879] | 1.000 | 0.0042 [0.0014,0.007] | 0.336 | 0.004 [0.0002,0.0079] | 1.000 |
| STAMBP | STAM-binding protein (STAMPB) | 0.73% | 0.0044 [0.0022,0.0066] | 0.009 | 0.0759 [-0.0586,0.2104] | 1.000 | 0.003 [0,0.0059] | 1.000 | 0.0044 [0.0006,0.0082] | 1.000 |
| TGF-alpha | Transforming growth factor alpha (TGF-alpha) | 0.73% | 0.0011 [-0.001,0.0033] | 1.000 | 0.1483 [0.0185,0.2782] | 1.000 | 0.003 [0.0003,0.0057] | 1.000 | -0.0024 [-0.0061,0.0013] | 1.000 |
| TNF | Tumor necrosis factor (TNF) | 0.73% | 0.0016 [-0.0006,0.0038] | 1.000 | 0.0224 [-0.1082,0.1531] | 1.000 | 0.0011 [-0.0015,0.0037] | 1.000 | 0.0025 [-0.0013,0.0063] | 1.000 |
| TNFB | TNF-beta (TNFB) | 0.73% | -0.0001 [-0.0023,0.0021] | 1.000 | -0.1416 [-0.2736,-0.0095] | 1.000 | -0.0005 [-0.0033,0.0023] | 1.000 | 0.0004 [-0.0034,0.0041] | 1.000 |
| TNFRSF9 | Tumor necrosis factor receptor superfamily member 9 (TNFRSF9) | 0.73% | 0.0003 [-0.0017,0.0022] | 1.000 | -0.0756 [-0.1931,0.042] | 1.000 | 0.0005 [-0.0017,0.0027] | 1.000 | -0.0004 [-0.0039,0.0031] | 1.000 |
| TNFSF14 | Tumor necrosis factor ligand superfamily member 14 (TNFSF14 ) | 0.73% | 0.0007 [-0.0015,0.0029] | 1.000 | 0.1659 [0.0344,0.2975] | 1.000 | 0.0012 [-0.0017,0.0041] | 1.000 | -0.0002 [-0.0039,0.0035] | 1.000 |
| TRAIL | TNF-related apoptosis-inducing ligand (TRAIL) | 0.73% | 0.0009 [-0.0013,0.003] | 1.000 | -0.1065 [-0.2351,0.0221] | 1.000 | -0.0007 [-0.0039,0.0024] | 1.000 | 0.0029 [-0.0005,0.0062] | 1.000 |
| TRANCE | TNF-related activation-induced cytokine (TRANCE) | 0.73% | -0.0017 [-0.0039,0.0004] | 1.000 | -0.1736 [-0.3037,-0.0436] | 0.832 | -0.003 [-0.006,0.0001] | 1.000 | 0.0001 [-0.0035,0.0037] | 1.000 |
| TSLP | Thymic stromal lymphopoietin (TSLP) | 93.17% |  |  |  |  |  |  |  |  |
| TWEAK | Tumor necrosis factor (Ligand) superfamily, member 12 (TWEAK) | 0.73% | -0.0025 [-0.0047,-0.0003] | 1.000 | -0.2266 [-0.357,-0.0963] | 0.064 | -0.0034 [-0.0066,-0.0002] | 1.000 | -0.0006 [-0.004,0.0029] | 1.000 |
| uPA | Urokinase-type plasminogen activator (uPA) | 0.73% | -0.0003 [-0.0025,0.0019] | 1.000 | -0.1213 [-0.2538,0.0112] | 1.000 | -0.0008 [-0.0042,0.0027] | 1.000 | -0.0001 [-0.0034,0.0032] | 1.000 |
| VEGFA | Vascular endothelial growth factor A (VEGF-A) | 0.73% | 0.0009 [-0.0012,0.003] | 1.000 | 0.172 [0.0477,0.2962] | 0.627 | 0.0017 [-0.0008,0.0042] | 1.000 | -0.0005 [-0.0041,0.0032] | 1.000 |

***Table S2:*** Median [95% CI)] values for each plasma protein in the total sample, and stratified for diabetes (yes/no). P values are calculated using Mann-Whitney-U-Tests.

| **Parameter** | **Total sample** | **Diabetes** | **No Diabetes** | **P value** |
| --- | --- | --- | --- | --- |
| 4E-BP1 | 8.7 [8.2-9.4] | 8.8 [8.4-9.5] | 8.7 [8.2-9.3] | 0.060 |
| ADA | 6.5 [6.2-6.8] | 6.4 [6.2-6.8] | 6.5 [6.2-6.8] | 0.763 |
| ARTN | 2.6 [2.2-3.0] | 2.6 [2.2-3.1] | 2.6 [2.2-3.0] | 0.992 |
| AXIN1 | 3.7 [3.4-4.2] | 3.9 [3.5-4.2] | 3.7 [3.3-4.1] | 0.105 |
| Beta-NGF | 0.2 [0.2-0.3] | 0.2 [0.2-0.3] | 0.2 [0.2-0.3] | 0.792 |
| CASP-8 | 3.3 [3.0-3.6] | 3.3 [3.0-3.7] | 3.3 [3.0-3.6] | 0.426 |
| CCL11 | 8.8 [8.4-9.2] | 8.8 [8.4-9.2] | 8.8 [8.4-9.2] | 0.799 |
| CCL19 | 9.1 [8.5-9.7] | 9.1 [8.7-9.9] | 9.1 [8.5-9.7] | 0.048 |
| CCL20 | 7.5 [6.9-8.2] | 7.6 [6.7-8.3] | 7.4 [6.9-8.1] | 0.670 |
| CCL23 | 11.2 [10.7-11.7] | 11.2 [10.8-11.9] | 11.1 [10.7-11.6] | 0.251 |
| CCL25 | 7.3 [6.9-7.8] | 7.4 [7.0-7.9] | 7.3 [6.9-7.8] | 0.065 |
| CCL28 | 5.9 [4.6-7.2] | 5.8 [4.3-7.3] | 5.9 [4.6-7.1] | 0.868 |
| CCL3 | 7.3 [6.8-7.7] | 7.3 [6.8-7.8] | 7.3 [6.8-7.7] | 0.524 |
| CCL4 | 7.6 [7.1-8.3] | 7.7 [7.0-8.4] | 7.6 [7.1-8.2] | 0.989 |
| CD244 | 6.6 [6.4-6.9] | 6.6 [6.3-6.9] | 6.6 [6.4-6.9] | 0.823 |
| CD40 | 11.9 [11.6-12.3] | 11.9 [11.6-12.3] | 11.9 [11.7-12.2] | 0.750 |
| CD5 | 6.7 [6.4-7.0] | 6.7 [6.4-6.9] | 6.7 [6.4-7.0] | 0.933 |
| CD6 | 6.4 [6.0-6.7] | 6.4 [6.0-6.7] | 6.4 [6.0-6.7] | 0.915 |
| CD8A | 10.2 [9.8-10.7] | 10.3 [9.9-10.8] | 10.2 [9.8-10.6] | 0.319 |
| CDCP1 | 4.0 [3.6-4.4] | 4.1 [3.6-4.5] | 3.9 [3.5-4.4] | 0.091 |
| CSF-1 | 10.8 [10.7-11.0] | 10.9 [10.7-11.1] | 10.8 [10.7-11.0] | 0.017 |
| CST5 | 6.4 [6.0-6.7] | 6.3 [6.0-6.7] | 6.4 [5.9-6.7] | 0.861 |
| CX3CL1 | 4.9 [4.5-5.2] | 4.8 [4.5-5.2] | 4.9 [4.6-5.2] | 0.682 |
| CXCL1 | 10.2 [9.8-10.6] | 10.3 [9.9-10.7] | 10.2 [9.8-10.6] | 0.060 |
| CXCL10 | 9.5 [9.0-10.1] | 9.6 [8.9-10.2] | 9.5 [9.0-10.1] | 0.956 |
| CXCL11 | 8.0 [7.4-8.6] | 8.1 [7.4-8.8] | 8.0 [7.5-8.6] | 0.481 |
| CXCL5 | 12.0 [11.2-12.6] | 11.9 [10.8-12.7] | 12.0 [11.2-12.5] | 0.303 |
| CXCL6 | 9.7 [9.2-10.1] | 9.7 [9.3-10.3] | 9.7 [9.2-10.1] | 0.192 |
| CXCL9 | 9.0 [8.2-9.9] | 9.1 [8.2-9.9] | 9.0 [8.2-9.9] | 0.612 |
| DNER | 9.3 [9.0-9.5] | 9.2 [9.0-9.4] | 9.3 [9.1-9.5] | 0.002 |
| EN-RAGE | 6.2 [5.6-6.9] | 6.4 [5.9-7.0] | 6.2 [5.5-6.9] | 0.207 |
| FGF-19 | 9.1 [8.4-9.8] | 8.8 [8.2-9.4] | 9.2 [8.5-9.9] | 0.004 |
| FGF-21 | 7.4 [6.4-8.4] | 7.6 [6.5-8.9] | 7.3 [6.4-8.3] | 0.11 |
| FGF-23 | 1.3 [1.0-1.8] | 1.4 [1.1-1.9] | 1.3 [1.0-1.7] | 0.080 |
| FGF-5 | 3.2 [2.9-3.8] | 3.2 [2.9-3.8] | 3.2 [2.9-3.9] | 0.851 |
| Flt3L | 9.7 [9.4-10.1] | 9.7 [9.3-10.1] | 9.7 [9.4-10.1] | 0.361 |
| GDNF | 3.0 [2.7-3.3] | 3.0 [2.6-3.4] | 3.0 [2.7-3.3] | 0.690 |
| HGF | 13.5 [12.6-13.9] | 13.7 [12.4-14.0] | 13.4 [12.7-13.8] | 0.181 |
| IFN-gamma | 6.6 [6.0-7.4] | 6.6 [5.9-7.6] | 6.6 [6.0-7.4] | 0.962 |
| IL-1 alpha | -1.1 [-1.3--0.9] | -1.1 [-1.3--0.9] | -1.1 [-1.3--0.9] | 0.627 |
| IL10 | 5.7 [4.9-6.7] | 5.7 [5.1-6.9] | 5.7 [4.9-6.7] | 0.549 |
| IL-10RA | 2.0 [1.9-2.3] | 2.0 [1.9-2.3] | 2.0 [1.9-2.3] | 0.337 |
| IL-10RB | 7.8 [7.6-8.0] | 7.8 [7.6-8.1] | 7.8 [7.6-8.0] | 0.695 |
| IL-12B | 7.6 [7.1-8.0] | 7.6 [7.2-8.1] | 7.6 [7.0-8.0] | 0.163 |
| IL13 | 1.2 [1.0-1.4] | 1.2 [1.0-1.6] | 1.2 [1.0-1.4] | 0.381 |
| IL-15RA | 2.7 [2.5-3.0] | 2.8 [2.5-3.0] | 2.7 [2.5-2.9] | 0.376 |
| IL-17A | 3.5 [3.2-3.9] | 3.6 [3.3-3.9] | 3.5 [3.2-3.8] | 0.379 |
| IL-17C | 3.9 [3.4-4.5] | 4.0 [3.4-4.7] | 3.9 [3.4-4.4] | 0.223 |
| IL18 | 9.9 [9.6-10.3] | 10.0 [9.7-10.4] | 9.8 [9.6-10.2] | 0.042 |
| IL-18R1 | 9.0 [8.7-9.3] | 9.1 [8.9-9.5] | 8.9 [8.6-9.2] | <0.001 |
| IL2 | 1.9 [1.7-2.0] | 1.8 [1.7-2.0] | 1.9 [1.7-2.0] | 0.600 |
| IL-20 | 1.8 [1.7-1.9] | 1.8 [1.7-1.9] | 1.8 [1.7-1.9] | 0.393 |
| IL-20RA | 2.1 [1.9-2.3] | 2.1 [1.9-2.3] | 2.1 [1.9-2.3] | 0.896 |
| IL-22 RA1 | 2.5 [2.2-2.7] | 2.5 [2.2-2.7] | 2.5 [2.2-2.7] | 0.575 |
| IL-24 | 2.7 [2.4-3.0] | 2.6 [2.4-3.0] | 2.7 [2.4-3.0] | 0.311 |
| IL-2RB | 2.0 [1.9-2.2] | 2.0 [1.9-2.2] | 2.0 [1.9-2.2] | 0.587 |
| IL33 | 2.1 [1.9-2.2] | 2.1 [1.9-2.2] | 2.1 [1.9-2.2] | 0.229 |
| IL4 | 1.9 [1.6-2.2] | 1.8 [1.6-2.2] | 1.9 [1.6-2.2] | 0.302 |
| IL5 | 1.7 [1.5-2.0] | 1.7 [1.5-2.0] | 1.7 [1.5-2.0] | 0.661 |
| IL6 | 5.6 [5.0-6.5] | 6.0 [5.2-7.4] | 5.5 [4.8-6.3] | <0.001 |
| IL7 | 4.4 [3.8-4.9] | 4.3 [3.7-4.8] | 4.4 [3.9-4.9] | 0.176 |
| IL8 | 6.1 [5.3-6.7] | 6.4 [5.4-7.4] | 6.0 [5.2-6.5] | 0.003 |
| LAP TGF-beta-1 | 8.3 [7.9-8.6] | 8.3 [7.9-8.7] | 8.3 [7.9-8.6] | 0.872 |
| LIF | 0.8 [0.6-1.1] | 0.9 [0.6-1.4] | 0.7 [0.5-1.0] | 0.007 |
| LIF-R | 4.4 [4.2-4.7] | 4.5 [4.3-4.8] | 4.4 [4.2-4.6] | 0.014 |
| MCP-1 | 12.7 [12.2-13.3] | 12.8 [12.1-13.3] | 12.7 [12.2-13.3] | 0.905 |
| MCP-2 | 10.3 [9.7-10.8] | 10.2 [9.6-10.6] | 10.3 [9.7-10.8] | 0.291 |
| MCP-3 | 2.6 [2.3-3.1] | 2.8 [2.4-3.4] | 2.6 [2.2-3.0] | 0.030 |
| MCP-4 | 13.9 [13.3-14.6] | 14.0 [13.4-14.6] | 13.9 [13.3-14.6] | 0.516 |
| MMP-1 | 15.4 [14.6-16.0] | 15.2 [14.4-15.8] | 15.5 [14.8-16.0] | 0.056 |
| MMP-10 | 9.8 [9.3-10.2] | 9.8 [9.4-10.2] | 9.7 [9.3-10.2] | 0.491 |
| NRTN | 2.1 [1.8-2.4] | 2.1 [1.9-2.4] | 2.1 [1.8-2.4] | 0.362 |
| NT-3 | 3.0 [2.8-3.2] | 3.0 [2.8-3.2] | 3.0 [2.8-3.3] | 0.150 |
| OPG | 11.2 [10.8-11.5] | 11.3 [11.0-11.8] | 11.1 [10.8-11.4] | 0.001 |
| OSM | 6.6 [5.9-7.5] | 6.6 [6.0-7.5] | 6.6 [5.9-7.4] | 0.589 |
| PD-L1 | 6.5 [6.3-6.8] | 6.5 [6.2-6.9] | 6.5 [6.3-6.8] | 0.743 |
| SCF | 9.7 [9.3-10.0] | 9.7 [9.1-10.0] | 9.7 [9.3-10.0] | 0.275 |
| SIRT2 | 4.1 [3.6-4.8] | 4.2 [3.8-4.8] | 4.1 [3.6-4.7] | 0.175 |
| SLAMF1 | 3.6 [3.3-4.0] | 3.7 [3.3-4.1] | 3.6 [3.3-3.9] | 0.107 |
| ST1A1 | 3.2 [2.6-3.8] | 3.0 [2.5-3.7] | 3.2 [2.6-3.9] | 0.254 |
| STAMBP | 5.0 [4.7-5.5] | 5.0 [4.7-5.5] | 5.0 [4.7-5.5] | 0.543 |
| TGF-alpha | 4.3 [3.8-5.1] | 4.3 [3.8-5.4] | 4.3 [3.8-5.1] | 0.389 |
| TNF | 4.5 [4.2-4.8] | 4.5 [4.2-4.8] | 4.5 [4.2-4.8] | 0.877 |
| TNFB | 5.2 [4.8-5.5] | 5.1 [4.6-5.4] | 5.2 [4.9-5.5] | 0.026 |
| TNFRSF9 | 6.7 [6.3-7.1] | 6.7 [6.3-7.1] | 6.7 [6.4-7.1] | 0.716 |
| TNFSF14 | 6.5 [6.0-7.3] | 6.5 [6.0-7.3] | 6.5 [6.0-7.2] | 0.758 |
| TRAIL | 8.3 [8.0-8.6] | 8.2 [7.8-8.5] | 8.4 [8.1-8.6] | 0.007 |
| TRANCE | 5.3 [4.8-5.6] | 5.3 [4.6-5.5] | 5.3 [4.9-5.6] | 0.066 |
| TSLP | 2.1 [1.8-2.3] | 2.0 [1.8-2.3] | 2.1 [1.8-2.3] | 0.282 |
| TWEAK | 11.1 [10.3-11.8] | 11.0 [9.8-11.9] | 11.2 [10.4-11.8] | 0.234 |
| uPA | 10.3 [10.1-10.6] | 10.3 [10.1-10.6] | 10.3 [10.1-10.5] | 0.465 |
| VEGFA | 11.6 [11.2-12.2] | 11.7 [11.3-12.4] | 11.5 [11.2-12.1] | 0.063 |
